# A High Refractive Index Plasmonic Micro-Channel Sensor Based on Photonic Crystal Fiber

**DOI:** 10.3390/nano12213764

**Published:** 2022-10-26

**Authors:** Jiangtao Lv, Tong Liang, Qiongchan Gu, Qiang Liu, Yu Ying, Guangyuan Si

**Affiliations:** 1 College of Information Science and Engineering, Northeastern University, Shenyang 110004, China; 2 Hebei Key Laboratory of Micro-Nano Precision Optical Sensing and Measurement Technology, Qinhuangdao 066004, China; 3 College of Information & Control Engineering, Shenyang Jianzhu University, Shenyang 110168, China; 4 Melbourne Centre for Nanofabrication, Victorian Node of the Australian National Fabrication Facility, Clayton 3168, VIC, Australia

**Keywords:** micro-channel, high analyte refractive index, confinement loss

## Abstract

A new concave shaped high refractive index plasmonic sensor with a micro-channel is proposed in this work, which comprises an analyte channel in the core hole. The sensor is elaborately designed to reduce the interference effect from the metal coating. Furthermore, the impact of the proposed structure on the sensitivity is also investigated by engineering the geometric parameters using the finite element method. We select gold as the plasmonic material in this theoretical study because it is widely used to fabricate plasmonic and metamaterial devices due to its chemical stability and compatibility. According to wavelength interrogation technique, simulations results show that this sensor can obtain maximal wavelength sensitivity of 10,050 nm/refractive index unit. In view of the excellent indicators of this device, it has important development potential in chemical and biological research fields.

## 1. Introduction

Surface plasmon resonance (SPR) can help detect environmental changes through evanescent waves [1]. SPR sensors play the most important role in sensing and monitoring food safety, drug development, environmental pollution, life science and public safety, etc. [2,3,4]. Kretschmann structure is commonly used in previous SPR sensor designs, which is characterized by depositing a metal layer on a prism substrate [5]. Kretschmann structure is a prism based configuration that forms SPR on the metal interface under the irradiation of light at a specific angle. However, the prism based SPR sensor cannot be further applied due to its structural limitations. Various fiber-based SPR sensors have advantages over prisms. However, among many optical fiber sensors, photonic crystal fiber (PCF) is preferred by researchers due to their high integration, design flexility, and great miniaturization potential [6,7].

In previous academic reports, precious metals such as gold (Au) and silver (Ag) with excellent plasmonic characteristics are applied for various SPR sensors. Although Ag provides high detection accuracy and sensitivities, Au exhibits better stability advantages. The internally metal-coated sensor is difficult to manufacture because it requires the metal and analyte to be filled into micron-sized air holes [8]. In contrast, the externally metal-coated PCF sensor is not only easy to manufacture, but also able to offer more design possibilities. The D-type optical fiber is a kind of non-circular symmetrical optical fiber structure, which is made by polishing off nearly part of the cladding on the side of the optical fiber. Researchers have found that the D-type fiber has a higher sensitivity and excellent structural performance [9,10,11,12,13]. However, the D-type fiber is also difficult to fabricate in the manufacturing processes. In addition to manufacturing difficulties, high refractive index (RI) detection is another obstacle of PCF sensors. In general, the detection range is usually less than 1.42 for most common PCF sensors [14,15,16]. In order to achieve high RI sensing, a hollow PCF fiber core can be filled with analyte to form a waveguide [17]. Therefore, it has great potential to design high RI sensors by using this method.

## 2. Materials and Methods

Here, we theoretically design a novel micro-channel assisted D-type hollow PCF-SPR sensor. The hollow core structure of the designed sensor can achieve high RI detection for liquid analytes which can be thoroughly verified by simulation results, and the addition of micro-channels in a D-type PCF can significantly reduce the difficulty of polishing. Moreover, the non-direct contact between the plasmonic materials and the analyte can further minimize the interference effects. As a result, the sensor shows the advantages of favorable sensitivity and real-time sensing in the performance measuring methods.

**Figure 1:**
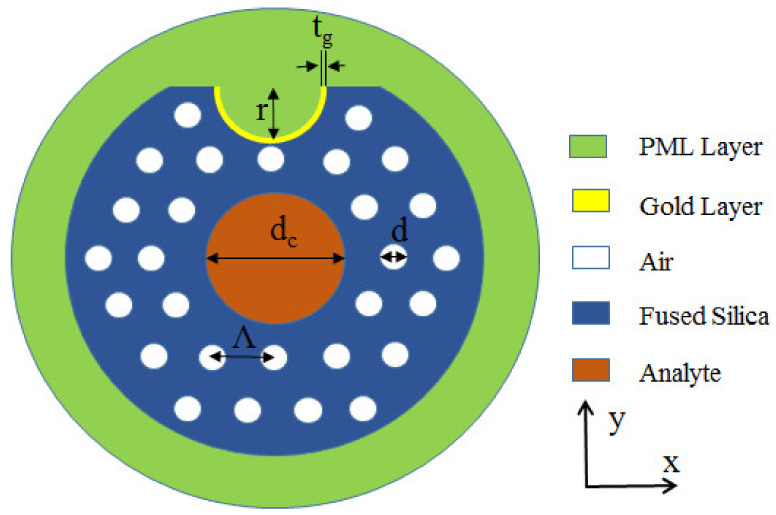
Geometry with labeled pore diameter (d), analyte-core diameter (d_c_), pore spacing (Λ), Au layer thickness (t_g_) and micro-channel radius (r).

The scheme of the concave shaped high RI plasmonic sensor is illustrated in Figure 1. The diameter of the analyte-core is much larger than the diameter of the surrounding pores. The micro-channel is designed on the polished surface and directly located above the core. Furthermore, the micro-channel is coated with Au, acting as the plasmonic material. With the help of COMSOL Multiphysics software, the sensing performance of the proposed device is simulated by finite element method. The whole model is constructed by using the super thinning standard of free triangle mesh. The complete mesh consists of 22,228 domain elements and 1246 boundary elements. The critical parameters are pore diameter (d), analyte-core diameter (d_c_), pore spacing (Λ), Au layer thickness (t_g_) and micro-channel radius (r). Au is evenly coated on the channel surface, which cannot only avoid direct contact with the analyte, but also facilitate real-time sensing. Moreover, absorbing light reflection can improve the accuracy of results through perfectly matched layer (PML). The RI of fused silica is calculated as follows [18,19,20,21]:

(1) $$n^{2}(\lambda )=1+\frac{B_{1}\lambda^{2}}{\lambda^{2}-{\;C}_{1}}+\frac{B_{2}\lambda^{2}}{\lambda^{2}-{\;C}_{2}}+\frac{B_{3}\lambda^{2}}{\lambda^{2}-{\;C}_{3}}$$

where B_1_ = 0.696166300, B_2_ = 0.407942600, B_3_ = 0.897479400, C_1_ = 4.6791482626 × 10^−3^ μm^2^, C_2_ = 1.35120631 × 10^−2^ μm^2^ and C_3_ = 97.9340025 μm^2^.

The confinement loss is evaluated as follows [22,23,24]:

(2) α(x,y)=8.686 × 2πλ × Im(neff) × 104

where λ is the wavelength in μm and Im(n_eff_) is the imaginary part of the effective RI.

## 3. Results and Discussion

**Figure 2:**
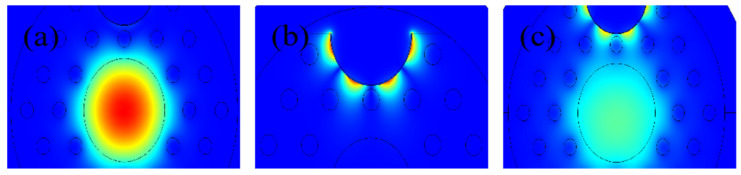
Electric field distribution showing optical coupling strength of (**a**) core mode, (**b**) SPP mode, and (**c**) the resonance coupling mode.

**Figure 3:**
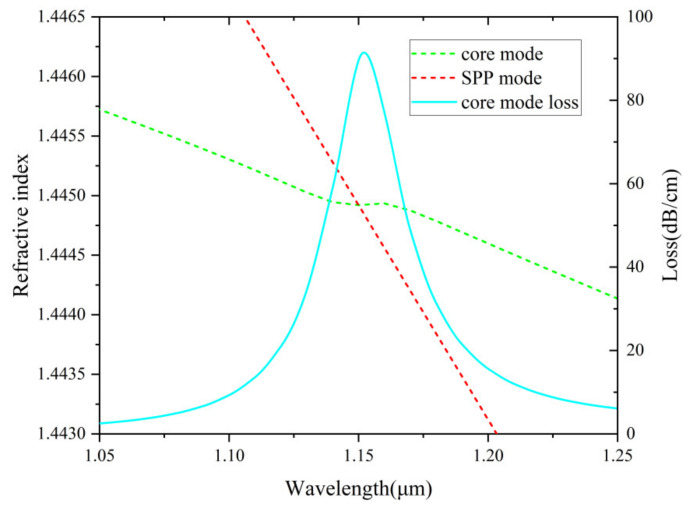
The change of loss spectrum and refractive index of the two modes (core and SPP) at n_a_ =1.45 as a function of wavelength.

The electric field distribution of the fundamental core mode, surface plasmon polariton (SPP) mode, and the resonance coupling mode of the proposed sensor is plotted in Figure 2. The structural parameters of electric field distribution simulation conditions are d_c_ = 5 μm, d = 0.7 μm, Λ = 2.1 μm, r = 2 μm and t_g_ = 30 nm. It can be seen clearly from Figure 2 that three fundamental modes reveal in (a), (b), and (c), respectively. Different fundamental modes lead to different energy distributions. Figure 3 illustrates the changes of loss spectra and RI of the two modes (core and SPP) when the analyte RI is 1.45. The curves of the two modes fall in a nearly straight line and intersect at 1.15 μm midway. At this resonance condition, the coupling between the two modes is the strongest, which leads to a sharp resonance peak.

**Figure 4:**
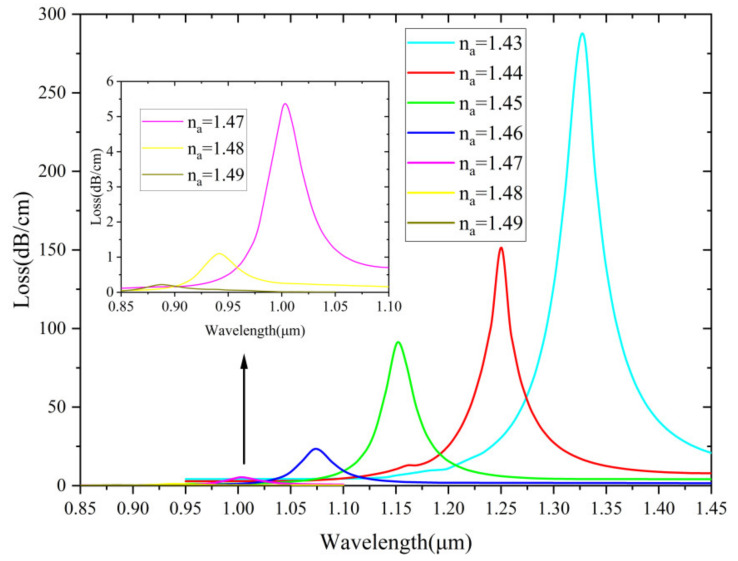
The loss spectrum of the core mode with different n_a_ from 1.43 to 1.49.

In Figure 4, the effect of loss spectra is also examined when the RI of analyte is varied from 1.43 to 1.49. As can be seen, when the n_a_ value is very high, the illustration shows that the peak loss value is extremely low. The loss depth represents the coupling degree of the core and SPP modes. Different n_a_ values lead to different resonance conditions so that the peak wavelength blue shifts. When n_a_ becomes higher, the loss spectra decrease rapidly. Figure 4 shows that the detection range of this sensor is higher than 1.42, which is excellent for most commonly used PCF sensors. This is because the analyte is placed in the fiber core to form a waveguide so that the analyte with a higher RI than the background material can be detected. 

The wavelength sensitivity is evaluated as follows [25]:

(3) $$S_{\lambda }(\mathrm{nm}/\mathrm{RIU})=\Delta \lambda_{\mathrm{peak}}/\Delta n_{a}$$

where Δ$\lambda_{\mathrm{peak}}$ indicates the resonance wavelength shifts and Δn_a_ represents the change of analyte RI. RIU represents refractive index unit.

Along with wavelength sensitivity, sensor resolution is considered as another important performance parameter. The sensor resolution is calculated as follows [26]:

(4) $$R(\mathrm{RIU})=\Delta n_{a}\Delta \lambda_{\min}/\Delta \lambda_{\mathrm{peak}}$$

where Δ$\lambda_{\min}$ = 0.1 nm (minimum spectral resolution). The maximum resolution of the proposed sensor is obtained as 9.95 × 10^−6^ RIU. Therefore, it shows that the measurement scale of the minute change in the RI of the analyte detected by the sensor is 10^−6^.

The figure of merit (FOM) is calculated as follows [27]:

(5) $$\mathrm{FOM}=S_{\lambda }/\mathrm{FWHM}$$

where FWHM is the full width at half maximum of the loss spectrum. 

**Figure 5:**
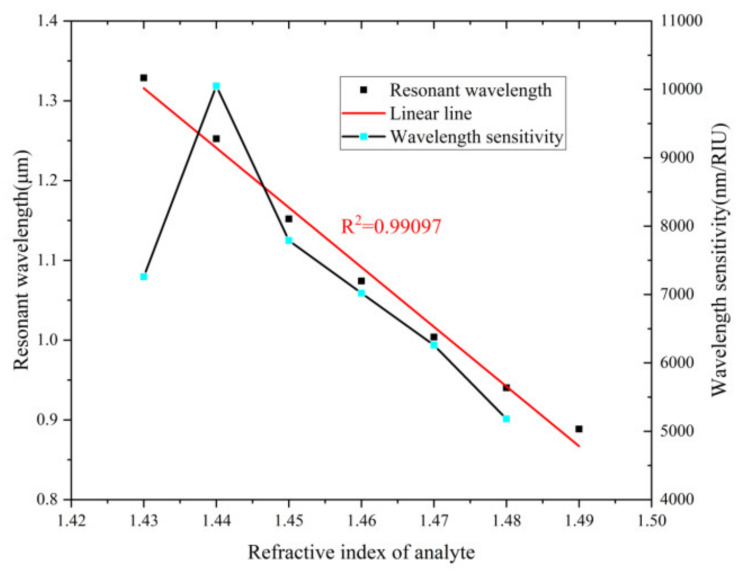
The resonance wavelength and wavelength sensitivity as a function of n_a_.

Figure 5 depicts the change trend of resonance wavelength and wavelength sensitivity. The sensor shows a good linearity (R^2^ is 0.99097) with analyte RI from 1.43 to 1.49. High linearity is regarded as a sign of a functional sensor. The highest sensitivity that can be obtained is 10,050 nm/RIU (n_a_ = 1.44). 

**Figure 6:**
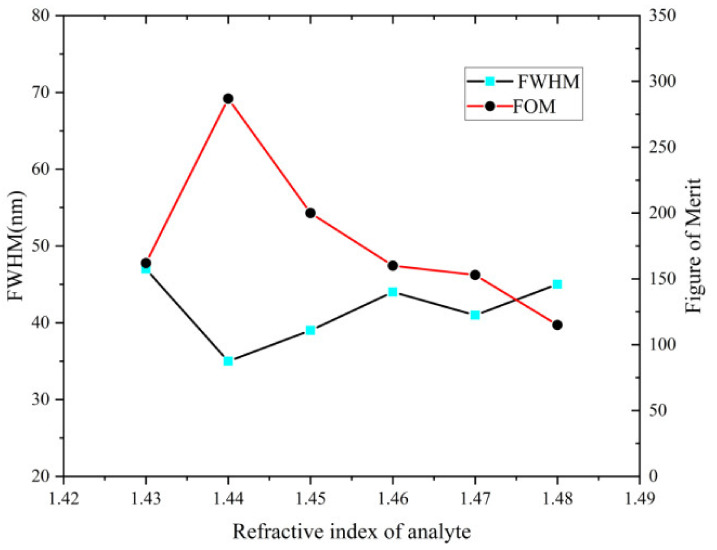
FWHM and FOM variation as a function of n_a_.

Figure 6 depicts the change trend of FOM and FWHM values with varying n_a_. The overall FOM shows a downward trend except for a brief rise from 1.43 to 1.44. The FWHM reveals the opposite trend, which is because these two parameters are reciprocal to each other. The sensor displays the highest value of FOM (287 RIU^−1^) with n_a_ = 1.44. 

**Figure 7:**
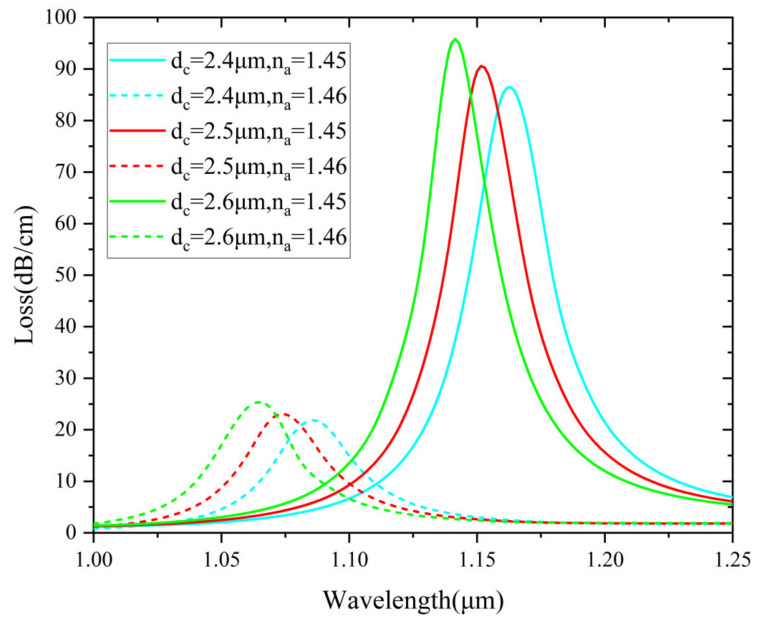
Loss spectra (n_a_ = 1.45 and 1.46) for different d_c_ of 2.4, 2.5 and 2.6 µm.

In order to find out the optimal structural parameters, analyte RI with 1.45 and 1.46 is further considered. Variation of the analyte-core diameter d_c_ is shown in Figure 7, for d_c_ values from 2.4 μm to 2.6 μm. Before optimizing d_c_, the other structural parameters remain unchanged. Increasing d_c_ can improve the resolution and obtain lower FWHM. According to the observation of the curves in the figure, the size of the analyte-core diameter has little interference with the sensor. Therefore, by taking into account the confinement loss value and low FWHM, d_c_ = 5 μm is considered as the optimized parameter.

**Figure 8:**
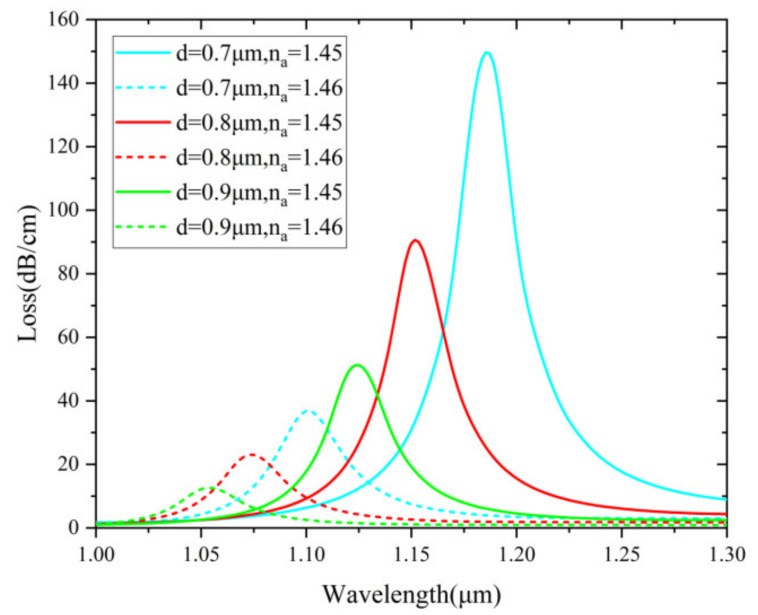
Loss spectra (n_a_ = 1.45 and 1.46) for different d of 0.7, 0.8 and 0.9 µm.

In the numerical simulations, the pore diameter d is also closely related to the confinement loss and resonant wavelength. Figure 8 illustrates the loss spectra of different d of 0.7, 0.8 and 0.9 µm. The resonant wavelength begins to shift rapidly as the pore diameter increases. It can be concluded that the loss spectra of different pore diameters vary obviously under different RI. Therefore, d = 0.7 μm is considered as the optimized value by taking into account both the sensitivity and FWHM.

**Figure 9:**
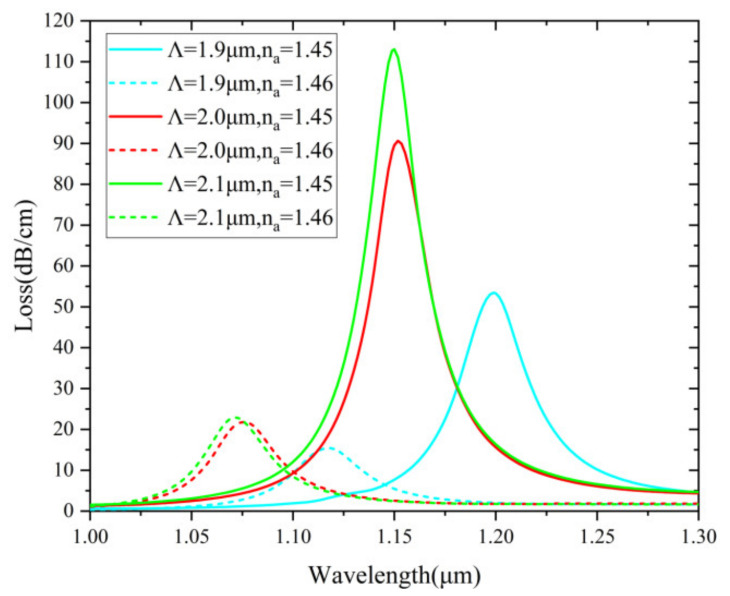
Loss spectra (n_a_ = 1.45 and 1.46) when Λ is varied from 1.9 to 2.1 µm.

In Figure 9, the effect of varying pore spacing is investigated. The resonant wavelength shifts dramatically first and then slowly as the pore spacing increases, which is because the phase-matching condition is changed. Moreover, with increasing pitch, the confinement loss increases accordingly. This is because the coupling strength with the plasmonic material is more intense after the spacing increases. Considering the strength of the coupling and sensing performances, Λ = 2.1 μm is determined as the optimized parameter.

**Figure 10:**
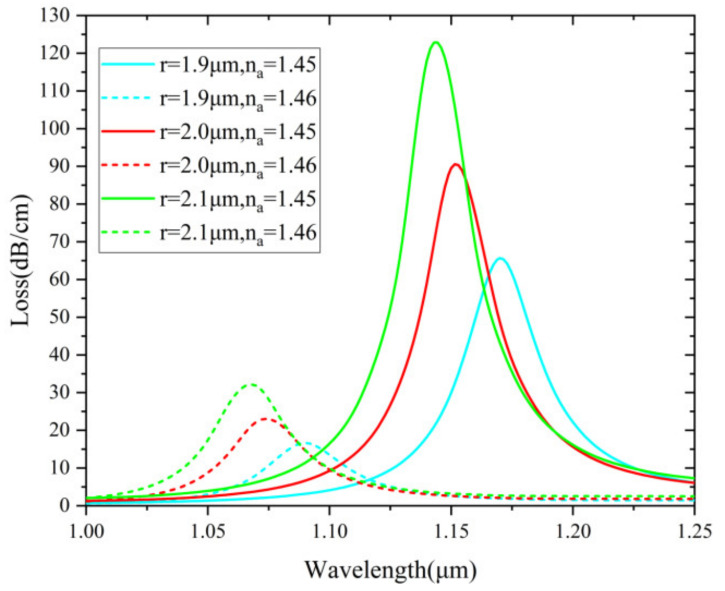
Loss spectra (n_a_ = 1.45 and 1.46) when r is varied from 1.9 to 2.1 µm.

**Figure 11:**
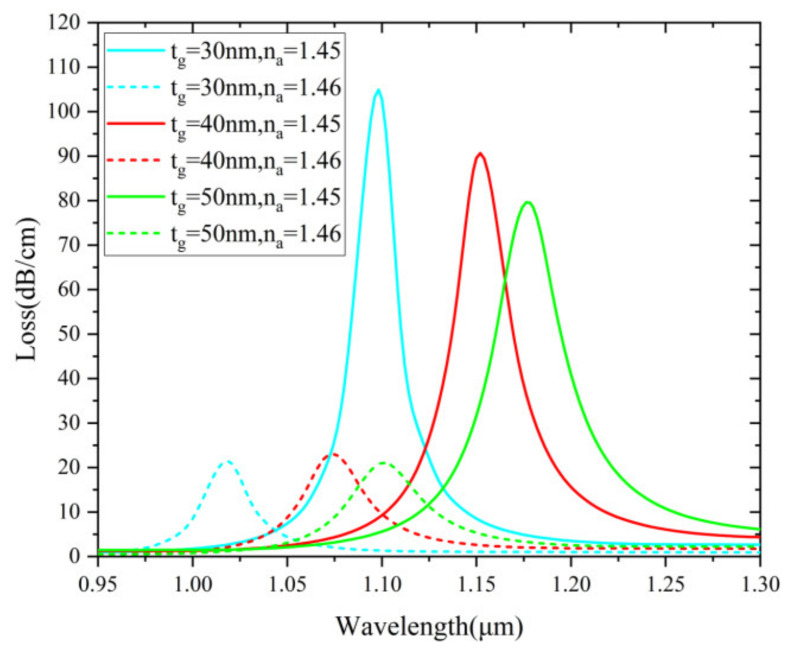
Loss spectra (n_a_ = 1.45 and 1.46) when t_g_ is varied from 30 to 50 nm.

In Figure 10, the effect of varying micro-channel radius (r) is examined from 1.9 μm to 2.1 μm. When r increases, SPR effect boosts. This results in the increase of the loss value. The wavelength sensitivities are 8000, 8400 and 8150 nm/RIU, respectively. The optimized value is considered as 2 μm by taking into account the sensing response stability. The proposed sensor is easier to design the thickness and location of the Au layer than most internally coated PCF sensors. This advantage makes the proposed sensor stand out and further enables fabrication possibilities using deposition and packaging techniques. Figure 11 shows loss spectra (n_a_ = 1.45 and 1.46) when t_g_ is varied from 30 to 50 nm (10 nm intervals). It is clearly observed that the resonant wavelength red shifts and loss depth reduces notably with increasing t_g_ for n_a_=1.45. The calculated wavelength sensitivities are 8150 nm, 8000 nm and 7900 nm/RIU, respectively. Considering the sensitivity and FWHM, t_g_ = 30 nm is determined as the optimized value.

## 4. Conclusions

In conclusion, we have designed a concave shaped high RI plasmon-assisted sensor with a micro-channel design. This work uses the finite element method to simulate the sensor and further characterize the performance. The proposed device has the special advantage of detecting high analyte RI and avoiding direct contact between analyte and the metal layer. The calculated results show that geometrical parameters possess a significant impact on device performances. The stack and draw technique can supports the drawing of the designed structure and chemical vapor deposition (CVD) or atomic layer deposition (ALD) can be used to deposit the Au layer for further packaging and integration. Under the optimized structural conditions, the proposed sensor demonstrates the highest sensitivity of 10,050 nm/RIU with a 9.95 × 10^-6^ RIU sensing resolution. The FOM of 287 RIU^−1^ and the high linearity of 0.99097 are realized with n_a_ from 1.43 to 1.49. The results show that the sensor has useful potential for high refractive index detection of chemical and biological liquids. Compared with the existing literature, the proposed sensor greatly simplifies the preparation process in PCF structure design and metal material coating, and improves the practical value. For the next step, further fabrication and experimental characterizations will be carried out. Moreover, dielectric materials with low optical losses are considered to replace the Au layer to further improve the sensing performance. In addition, the structural parameters are simulated within ±2% of the manufacturing tolerance of the optimized value, enabling further analysis of the device performance. 

## Data Availability

Not applicable.
